# The Effects of Tobacco Use During and After Pregnancy on Exposed Children: Relevance of Findings for Alcohol Research

**Published:** 2000

**Authors:** Marie D. Cornelius, Nancy L. Day

**Affiliations:** Marie D. Cornelius, Ph.D., is an associate professor of psychiatry and epidemiology and Nancy L. Day, Ph.D., is a professor of psychiatry, epidemiology, and pediatrics at the University of Pittsburgh School of Medicine, Pittsburgh, Pennsylvania

**Keywords:** tobacco in any form, smoking, pregnancy, adverse drug effect, postnatal AOD (alcohol or other drug) exposure, prenatal AOD exposure, infant, cognitive development, psychobehavioral AODE (effects of AOD use, abuse, and dependence), growth and development

## Abstract

Alcohol and tobacco use during pregnancy have both been associated with a number of adverse effects on the growth, cognitive development, and behavior of the exposed child. Understanding the effects of prenatal tobacco exposure allows researchers to identify those characteristics that are uniquely related to tobacco and those that are affected by alcohol exposure. This research, along with studies on the effects of alcohol use during pregnancy, has implications for preventing various types of substance use during pregnancy and for treating children affected by prenatal substance use.

Women who smoke during pregnancy are also likely to drink alcohol. In one survey, conducted as part of the Maternal Health Practices and Child Development (MHPCD) project in Pittsburgh, Pennsylvania, 76 percent of adult women who reported smoking during their first trimester of pregnancy said that they also drank alcohol during that period (Day et al. 1992). Among pregnant teenagers surveyed, 61 percent of those who smoked during the first trimester also drank alcohol (Cornelius et al. 1995). In addition, tobacco and alcohol use are both prevalent among women who use illicit drugs during pregnancy. In the National Pregnancy and Health Survey (National Institute on Drug Abuse [NIDA] 1996), 74 percent of women who used illicit drugs during pregnancy also reported either smoking, drinking, or both. The use of either one of these drugs is, in itself, a risk factor for poorer pregnancy outcome.

Although alcohol and tobacco are frequently used together during pregnancy, researchers studying the negative effects of prenatal exposure to tobacco and alcohol have generally examined the effects of each drug separately. Therefore, it is difficult to discuss the effects of the combined use of the two drugs. Although the other articles in this issue examine the use of alcohol and tobacco together, this article focuses on tobacco use during pregnancy and the effects of prenatal tobacco exposure. Understanding the effects of pre-natal tobacco exposure allows the identification of those characteristics that are uniquely related to tobacco and those that are affected by alcohol exposure. This research, along with research on the effects of alcohol use during pregnancy, has implications for preventing various types of substance use during pregnancy and for treating children affected by prenatal substance use.

Prenatal tobacco exposure has been reported to be a significant risk factor for sudden infant death syndrome (SIDS) (National Cancer Institute [NCI] 1999) and is estimated to be responsible for up to 4,800 infant deaths as well as 61,000 low-birth-weight (LBW) infants and 26,000 infants requiring neonatal intensive care annually (DiFranza and Lew 1995). In a national survey of pregnant adult women, however, 20.4 percent reported smoking cigarettes during pregnancy (NIDA 1996). This proportion rises to about one-half for women in lower socioeconomic populations (Cornelius et al. 1995; Day et al. 1992).

Smoking during pregnancy is more prevalent among Caucasian women compared with African-American or Hispanic women (NIDA 1996). Caucasian women also smoke at higher levels than do women of other ethnicities. Women who smoke during pregnancy are less likely to be married, have less education, have lower incomes, and attend fewer prenatal visits compared with women who do not smoke during pregnancy (Day et al. 1992; Cornelius et al. 1994).

Compared with alcohol, marijuana, and other illicit drug use, tobacco use is less likely to decline as the pregnancy progresses (Day et al. 2000; Cornelius et al. 1995). In the National Pregnancy and Health Study (NIDA 1996), approximately two-thirds of the women who smoked prior to their pregnancy continued smoking into the last trimester. In contrast, only one-fourth of the women who used alcohol prior to conception continued to drink into the third trimester. Women who smoke during pregnancy also continue smoking after the pregnancy (Cornelius et al. 1999*a*; Leech et al. 1999). Therefore, children born to women who use tobacco during pregnancy are likely to continue to be exposed to tobacco after birth. This environmental, or passive, exposure may also affect the children’s development.

## Effects of Smoking During Pregnancy

This section of the article reviews findings on the effects of maternal smoking during pregnancy on the exposed children’s growth, cognitive function, and behavior. The subsequent section focuses on the effects of passive smoking. Because of space limitations, this article does not review research on the effects of prenatal alcohol exposure. Because women who smoke during pregnancy are also likely to drink alcohol and use other drugs, many of the studies reviewed here controlled for prenatal alcohol exposure and other confounding factors, to determine the unique effects associated with prenatal tobacco exposure.

### Effects on Infant Growth

Maternal smoking during pregnancy has long been considered an important risk factor for LBW. This association was first reported in 1957 and has been proven in numerous subsequent studies (Stillman et al. 1986; U.S. Department of Health and Human Services [USDHHS] 1980; Floyd et al. 1993). Birth weight decreases in direct proportion to the number of cigarettes smoked (Persson et al. 1978; Yerushalmy 1971), and children of smokers are 150 to 250 grams lighter than are the children of nonsmokers (USDHHS 1980). The reduction in infant weight is not attributable to earlier gestation, because infants of smokers exhibit growth retardation at all gestational ages (NCI 1999). In a recent study of neonatal body composition, prenatal tobacco exposure was significantly related to having less fat-free mass, as measured by total body electrical conductivity (Lindsay et al. 1997). The authors concluded that the LBW of infants exposed to prenatal smoking is primarily attributable to reduced fat-free mass or lean tissue. Birth length and head and chest circumference are also reduced in infants who are prenatally exposed to tobacco (Cornelius et al. 1995; Day et al. 1992; Lindsay et al. 1997; Luciano et al. 1998).

In a recent study of pregnant teenagers (Cornelius et al. 1995, 1999*b*), more than one-half of whom were smokers, prenatal tobacco exposure was significantly related to reduced birth weight, birth length, head circumference, and chest circumference. These reductions were even more pronounced than those found in a similar cohort of the children of adult women (Day et al. 1992). For example, in the study of adult mothers and their children, prenatal tobacco use was significantly associated with a reduction in birth weight of 158 grams per pack per day. In the children of teenage mothers, prenatal tobacco exposure was significantly associated with a reduction in birth weight of 202 grams per pack per day. The increased problems associated with young maternal age and poor fetal outcomes (Fraser et al. 1995; Ketterlinus et al. 1990), coupled with the high prevalence of smoking among pregnant teenagers (Cornelius et al. 1994), magnify the risks to children of pregnant teenagers who smoke.

In another recent study on prenatal tobacco exposure and fetal growth, Zaren and colleagues (2000) reported that the male fetus might be more adversely affected than the female fetus. In this study, fetuses of nonsmoking, light smoking, and heavy smoking mothers were measured by sonograms at weeks 17, 25, 33, and 37. Boys born to heavy-smoking mothers had greater weight reductions, lower fat accretions, and smaller head circumferences when compared with girls of heavy smoking mothers.

Two key ingredients of cigarette smoke that are known to affect fetal growth are carbon monoxide and nicotine. Carbon monoxide causes fetal hypoxia, a reduction in the amount of oxygen available to the fetus (USDHHS 1980; Lambers and Clark 1996), whereas nicotine can lead to a decrease in the flow of oxygen and other nutrients across the placenta by constricting uterine arteries (Lambers and Clark 1996). In addition, nicotine itself can cross the placenta to affect the fetal cardiovascular and central nervous systems (CNS) (Stillman et al. 1986). Other constituents of tobacco smoke (e.g., cadmium and toluene) have also been shown to cause fetal growth retardation (Office of Environmental Health Hazard Assesment [OEHHA] 1996).

### Long-Term Effects on Growth

The effects of prenatal tobacco exposure on older children’s growth are not as clear as the effects on infants. Using data from the Collaborative Perinatal Project, Naeye (1981) detected a small difference in height and head circumference in exposed children at age 7. Rantakallio (1983) found that exposed children were shorter than nonexposed children at age 14, and Fogelman and Manor (1988) reported decreased height at ages 7, 11, and 23. In the latter study, the differences in height at age 23 were mediated by birth weight. These studies did not control for passive exposure to tobacco smoke or exposure to alcohol. A study of 714 three-year-old children found that the children of women who quit smoking during pregnancy were heavier and taller than those of women who did not quit (Fox et al. 1990). Adjustment for postpartum exposure to tobacco smoke reduced the difference in the children’s weight, but had little effect on differences in height.

Other studies have not found growth retardation over the long-term (Fried and O’Connell 1987; Hardy and Mellitus 1972). In addition, one study that followed infants from birth through 6.5 months and 13 months found that pre-natal alcohol exposure, rather than tobacco exposure, was associated with a slower growth rate when the exposed children were compared with unexposed children during the first 6.5 postpartum months. Although maternal smoking was correlated with shorter stature at 6.5 and 13 months, this effect was attributable to maternal drinking during pregnancy (Jacobson et al. 1994), highlighting the importance of controlling for the effects of other drugs.

The MHPCD study of adult mothers and their children (Day et al. 1992), which controlled for prenatal alcohol and other drug exposures and current maternal tobacco use, found a significant inverse relationship between maternal tobacco use during pregnancy and the infant’s weight, length, and head circumference at birth. At 8 months, only the infant’s length continued to be associated with prenatal tobacco exposure. When the children were followed up at 18 months and 6 years, prenatal tobacco exposure was not related to any growth reductions after controlling for the appropriate covariates (Day et al. 1994). Similarly, Vik and colleagues (1996) found that the reductions in birth weight that were attributed to pre-natal tobacco exposure were not evident when the children were 5 years old.

Prenatal tobacco exposure may not only be related to size deficits at birth, but may also be associated with disproportionate weight (for height) among both infants and young children. For example, a recent study of more than 200,000 births in Sweden found that prenatal tobacco exposure was significantly associated with reduced birth length and birth weight (Lindley et al. 2000). However, maternal smoking was also significantly associated with an increase in ponderal index, an indication of higher proportionate weight for height, when birth weight and gestational age were controlled for. Thus, the children of smokers tended to be shorter and have a higher ponderal index than children of nonsmokers. This finding is consistent with studies that have followed children after infancy. For example, Fried and colleagues (1999) found that prenatal tobacco exposure was related to an increased rate of obesity among 6-year-olds. The researchers proposed that this association was attributable to a preference for bottle-feeding among mothers who smoked during pregnancy. Vik and colleagues (1996) also reported a higher ponderal index and increased skinfold thickness (a measure of percentage body fat) in children whose mothers smoked during pregnancy, compared with children whose mothers did not smoke. Researchers evaluating the MHPCD cohort of 6-year-old children of teenage mothers also found a positive association between prenatal tobacco exposure and increased skinfold thickness. Prenatally exposed children also had higher values on the body mass index and weight-for-height Z-scores, an indication that the children were overweight for their height (Cornelius et al. in press*b*). Bottle-feeding was not a significant factor. Thus, several recent studies indicate that prenatal tobacco exposure seems to alter the relationship between body length and weight. This finding is underscored by two studies that have found that prenatal tobacco exposure reduces the growth of the long bones in the fetus (Lindsay et al. 1997; Luciano et al. 1998).

### Effects on Cognitive Function

Laboratory research with animals has shown that nicotine affects the CNS at exposure levels below those at which growth changes are evident (Slotkin 1998). For example, animal studies have shown associations between fetal nicotine exposure and increased locomotor activity in male rat pups (Shacka et al. 1997); hyperactivity in rats associated with increased nicotine receptors in the brain (Tizabi et al. 1997); lower turnover of the brain chemicals dopamine and serotonin in the rat brain as a result of alterations in the release or removal of dopamine and serotonin from the synapse (Muneoka et al. 1997); and changes in the morphology of the hippocampus in rats (Roy and Sabherwal 1998).

In the literature on humans, prenatal tobacco exposure has also been linked to CNS effects, including cognitive and neurobehavioral outcomes, although the reports are inconsistent. At birth, prenatal tobacco exposure has been associated with poorer auditory orientation and autonomic regulation (Picone et al. 1982) and increased tremors and startles (Fried and Makin 1987). In a recent race-matched study of cocaine-exposed and non-cocaine-exposed infants, neurological exams showed that prenatal tobacco exposure was significantly related to muscle tone abnormalities when controlling for other variables, including prenatal cocaine and ethanol exposure, head circumference, and prenatal care (Dempsey et al. 2000). The authors concluded that maternal cigarette smoking, rather than cocaine exposure, might be the major predictor of tone abnormalities.

Studies have also reported adverse effects of prenatal tobacco exposure on cognitive and behavioral development in older children. In one study, cognitive functioning at age 3 was higher among the children of mothers who quit smoking during pregnancy than among children whose mothers smoked throughout pregnancy (Sexton et al. 1990). Poor language development and lower cognitive scores have also been reported in 2- (Fried and Watkinson 1988), 3-, and 4-year-old (Fried and Watkinson 1990) children prenatally exposed to tobacco. When those children were 9 to 12 years old, prenatal tobacco exposure was negatively associated with language and reading abilities (Fried et al. 1997). In another analysis of this same cohort of 9 to 12 year-olds, prenatal tobacco exposure had a negative, dose-dependent association with visual perception after consideration of other potential prenatal risk factors and of pre- and post-natal secondhand smoke exposures (Fried and Watkinson 2000).

Other researchers (Baghurst et al. 1992; Fergusson and Lloyd 1991) have argued that initially significant associations between prenatal tobacco exposure and cognitive development were explained better by differences in social class and the home environment. For example, after controlling for socioeconomic and environmental differences, Eskenazi and Trupin (1995) failed to find consistent relationships between prenatal tobacco exposure and performance on the Raven Colored Matrices Test (Raven et al. 1986), a measure of nonverbal reasoning, or the Peabody Picture Vocabulary Test (PPVT) (Dunn and Dunn 1981). However, in the MHPCD study of adult mothers, prenatal tobacco exposure predicted deficits in visual memory and verbal learning scores on the Wide Range Assessment of Memory and Learning test (WRAML) (Sheslow and Adams 1990) (Cornelius et al. 1999*c*), and these associations remained after consideration of other factors, including socioeconomic status, maternal psychological status, home environment, other prenatal substance exposures, and current maternal tobacco and other substance use.

### Effects on Activity, Attention, and Impulsivity

Researchers have also reported associations between prenatal tobacco exposure and increased activity, inattention, and impulsivity. Streissguth and colleagues (1984) reported significant relationships between prenatal tobacco exposure and errors of omission and commission, reflective of inattention and impulsivity, respectively, in 4-year-olds. Kristjansson and colleagues (1989) found that prenatal tobacco exposure predicted impulsivity and increased overall activity among 4-to 7-year-olds after controlling for prenatal exposure to other drugs and post-natal exposure to second-hand smoke. In addition, Fried and colleagues (1992) reported a significant relationship between prenatal tobacco exposure and impulsivity among 6-year-olds in the same cohort.

Milberger and colleagues (1996) found a positive relationship between maternal smoking during pregnancy and an increased risk of attention deficit hyperactivity disorder in exposed children between the ages of 6 and 17, although the study did not control for current maternal smoking or prenatal exposure to other substances. In the MHPCD study of adult mothers, prenatal tobacco exposure significantly predicted increased errors of commission on the Continuous Performance Test (Lindgren and Lyons 1984) among 6-year-olds (Leech et al. 1999). However, the mothers’ current tobacco use correlated so highly with the prenatal exposure levels that these exposures could not be separated. Eskenazi and Trupin (1995) did not find a relationship between prenatal tobacco use and activity.

When the children of the adult mothers in the MHPCD study were assessed at age 10, prenatal tobacco exposure predicted deficits on neuropsychological tests that measured planning ability and fine motor coordination (Cornelius et al. 1999*c*). These deficits persisted after controlling for maternal current smoking, prenatal exposure to other substances, and covariates of prenatal and current substance use.

### Behavioral and Psychological Effects

Behavioral and psychological problems have also been linked to prenatal tobacco exposure. Orlebeke and colleagues (1997) reported a significant effect of prenatal tobacco exposure on externalizing behaviors, including oppositional, aggressive, and overactive behaviors in 3-year-olds. This study did not control for other prenatal substance exposures or the mothers’ current smoking habits. Weitzman and colleagues (1992) found that women who smoked both during and after pregnancy rated their children as having more behavior problems, but the researchers found no effects on children who were only exposed during pregnancy. Brook and colleagues (2000) found that mothers who smoked during pregnancy were significantly more likely to have toddlers who displayed negativity than did mothers who only smoked after delivery. This relationship was maintained after controlling for a number of psychosocial risk factors, including the mother’s distress, socioeconomic status, and perinatal risk factors. In the adult cohort of the MHPCD project, 3-year-olds who were exposed prenatally to tobacco were significantly more likely to display oppositional behavior, immaturity, and aggressive behavior, according to the mothers’ reports (Day et al. 2000). These relationships persisted after controlling for socioeconomic status, current home environment, maternal psychological status, current maternal tobacco use, and other prenatal substance exposures.

The behavior problems observed in toddlers prenatally exposed to tobacco persist through the adolescent and adult years. Fergusson and colleagues (1993) followed a birth cohort through age 12 and reported that prenatal tobacco exposure was significantly related to childhood behavior problems, whereas current maternal smoking was not. At ages 16 to 18, children in that cohort who were exposed to prenatal smoking had higher rates of conduct disorder, substance use, and depression than did nonexposed children (Fergusson et al. 1998). Wakschlag and colleagues (1997) also reported a significant relationship between prenatal tobacco exposure and conduct disorder in a clinical sample; however, this study did not control for current exposure. In addition, maternal smoking during pregnancy predicted persistent criminal outcomes in adult male offspring in a Danish prospective study (Brennan et al. 1999). That study controlled for a number of demographic variables, but it did not control for prenatal alcohol and illicit drug exposure or for environmental tobacco exposure. In another prospective study in Finland (Rasanen et al. 1999), maternal smoking during pregnancy was significantly associated with an increase in violent offenses among the adult male offspring.

A few studies have evaluated the relationships between prenatal substance exposure and subsequent substance use in the offspring. Animal researchers have noted that changes resulting from prenatal nicotine exposure might affect susceptibility to later tobacco use (Miao et al. 1998; Nordberg et al. 1991; Smith et al. 1991). In a retrospective study of humans, Kandel and colleagues (1994) reported a fourfold increased risk of tobacco use among female offspring who were exposed to tobacco prenatally. In a later report, Griesler and colleagues (1998) showed that maternal smoking during pregnancy was significantly associated with higher levels of child behavior problems and that these behavior problems increased the likelihood of smoking among daughters between the ages of 9 and 17. The association between prenatal tobacco exposure and early tobacco experimentation was also found in the MHPCD prospective study of adult women and their offspring (Cornelius et al. 2000). In this study, 10-year-old children exposed to tobacco at the level of at least one half pack per day during gestation had a 5.5-fold increased risk for early tobacco experimentation, controlling for prenatal exposure to other substances and their mothers’ current smoking habits.

## Effects of Prenatal Exposure to Environmental Tobacco Smoke

Pregnant women who do not smoke but live with or spend time with smokers expose their children to environmental tobacco smoke (ETS). In a review of 25 epidemiological studies of the relationship between fetal growth and ETS exposure, all but one study reported a decrement in mean birth weight with ETS exposure (NCI 1999). Martin and Bracken (1986) found that passive exposure to smoking during pregnancy was significantly correlated with lower birth weight among the children of nonsmoking women. Full-term newborns exposed only to passive smoke weighed 61 grams less than newborns not exposed to passive smoke and had a significantly increased risk of LBW. Data from the National Health Interview Survey showed that after controlling for potential confounding variables—including race, number of children (i.e., parity), income, and maternal age—nonsmoking women with high exposure to passive smoke were 1.6 times more likely to have a LBW infant than were nonsmokers with low exposure (Mainous and Hueston 1994).

Studies using biomarkers to measure passive exposure provide further evidence of an adverse effect on growth. For example, one study examined levels of cotinine (a product of nicotine metabolism) in pregnant nonsmokers (Haddow et al. 1988) and found that the infants of the passively exposed mothers weighed 108 grams less than infants of unexposed women, even after controlling for known birth-weight-associated covariates. Other studies have confirmed these findings (Eskenazi et al. 1995; Rebagliato et al. 1995).

Makin and colleagues (1991) examined the long-term effects of prenatal passive exposure on 6- to 9-year-olds and found that children of nonsmoking mothers generally performed better on tests of speech and language skills, intelligence, and visual-spatial abilities as well as on the mothers’ ratings of behavior, compared with children whose mothers were active or passive smokers. The performance of children of passive smokers was found, in most areas, to be between that of the children of active smokers and nonsmokers.

## Effects of Postnatal Exposure to Environmental Tobacco Smoke

Postnatal exposure to ETS has been significantly associated with an increased risk of SIDS. After considering the effects of socioeconomic status, prenatal care, prenatal tobacco exposure, birth weight, breast feeding, and routine infant sleeping position, Klonoff-Cohen and colleagues (1995) reported that children exposed to the smoking of more than 1 pack of cigarettes per day in a household were 22.7 times more likely than other children to develop sudden infant death syndrome (SIDS). Blair and colleagues (1996) reported that the risk of SIDS increased with the number of cigarettes smoked per day in the household, ranging from 2.5 for 1 to 19 cigarettes per day to 7.6 for 40 or more cigarettes per day.

Cognitive and behavioral outcomes are also affected by postnatal exposure to passive smoke. Postnatal exposure to household smoke was reported to be associated with reduced IQ scores in 3-year-olds (Johnson et al. 1993). However, the effects of prenatal exposure were not considered in this analysis. Eskenazi and Trupin (1995) reported that 5-year-olds who were environmentally exposed to tobacco smoke had significantly lower scores on the Raven test and PPVT and were rated as more active by their mothers. That analysis controlled for prenatal tobacco exposure.

Among 6- to 11-year-old children of nonsmoking mothers, McCartney and colleagues (1994) found that postnatal passive tobacco exposure resulted in scores on central auditory processing tasks that were similar to scores for children of mothers who were light smokers during pregnancy. In the MHPCD cohort of adolescent mothers and their 6-year-old children, passive tobacco exposure, measured by the child’s urine cotinine levels, was significantly related to poorer scores on subtests of the Test of Language Development (TOLD) (Hammill and Newcomer 1999), thereby reflecting lower receptive language abilities (Cornelius et al. in press*a*).

## Summary and Conclusions

Smoking during pregnancy has been associated significantly with a number of adverse effects on the growth, cognitive development, and behavior of the exposed child. However, because women who smoke during pregnancy are also likely to use alcohol or other drugs, researchers must account for these confounding factors in order to identify accurately the specific and unique role of tobacco exposure. In addition, even nonsmoking mothers can expose their children through environmental tobacco exposure. Compared with alcohol and other drug use, tobacco use is less likely to decline during pregnancy, and women who smoke during pregnancy are more likely to continue to smoke after delivery. This means that children who are prenatally exposed to tobacco are at higher risk for continued exposure to environmental tobacco smoke from the mother and from other household smokers.

Prenatal tobacco exposure has consistently been associated with deficits in weight, height, and head circumference at birth. In general, long-term studies that have controlled for other factors that affect growth, particularly prenatal alcohol exposure, have reported that these growth deficits are corrected by “catch-up” growth in early childhood. Other studies have indicated a disproportionate weight-to-height ratio with higher ponderal indices in infants and body mass indices in older children.

Recent research has noted a higher rate of behavioral problems among children who were prenatally exposed to tobacco. Higher rates of cognitive deficits, specifically in language, reading, and vocabulary, as well as poorer performances on tests of reasoning and memory have been reported. Researchers have also reported behavior problems, such as increased activity, inattention, impulsivity, opposition, and aggression. In addition, prenatal tobacco exposure has been associated with higher rates of delinquency and criminality in adolescence and adulthood, an outcome that is perhaps mediated by earlier behavior problems.

Heredity is an important factor to consider in the interpretation of these findings (Heath et al. 1995). A shared genetic component between the mother and child may represent a vulnerability to the characteristics that are associated with tobacco use. For example, prenatal tobacco exposure may result in more impulsive offspring, or women who are impulsive may be more likely to smoke and to produce more impulsive children. Data from animal studies demonstrate specific CNS changes resulting from prenatal nicotine exposure that may affect offspring behavior. Therefore, both genetic and teratological etiologies are most likely involved in the observed adverse outcomes.

The neurobehavioral effects of prenatal and postnatal tobacco exposure are subtle. They are not easily identified, and difficulties arise in demonstrating that many of the outcomes discussed in this review are “caused by” tobacco exposure (Ramsay and Reynolds 2000). Nevertheless, we must continue identifying both short- and long-term effects of prenatal and postnatal tobacco exposure on the growth and neurobehavioral development of children. Future studies should consider the separate and combined effects of tobacco and alcohol exposure on the developing offspring and use study designs and methodologies that allow researchers to tease out the effects from both prenatal and postnatal time periods. Without an understanding of the exact mechanisms by which tobacco exposure affects the CNS, the causes of the cognitive and behavior problems associated with prenatal tobacco exposure will possibly be attributed to another exposure, to environmental factors, or to the character of the mother or child.

Recognition and clarification of the effects of tobacco exposure on the development of the child may also help improve understanding of the effects of prenatal exposure to alcohol and other drugs. In addition, this research will facilitate the development of interventions to prevent substance use during pregnancy and to treat children prenatally exposed to tobacco, alcohol, and other drugs.
